# Associations of smartphone use duration and loneliness with depressive symptoms among Korean adolescents: a nationally representative cross-sectional study

**DOI:** 10.3389/fpubh.2026.1834994

**Published:** 2026-05-05

**Authors:** Guifang Liu, Kyung-Hee Lee

**Affiliations:** 1 School of Physical Education, Zhengzhou Normal University, Zhengzhou, China; 2 Department of Exercise Therapy, Graduate School of Special Therapy, Gachon University, Seongnam-si, Republic of Korea

**Keywords:** adolescents, cross-sectional study, depressive symptoms, mental health, perceived loneliness, smartphone use

## Abstract

**Background:**

Adolescent depression is a major public health concern, yet the relative contributions of smartphone use duration and perceived loneliness remain unclear. This study examined their independent and comparative associations with depressive symptoms in a nationally representative sample of Korean adolescents.

**Methods:**

This cross-sectional study analyzed data from 54,653 middle and high school students from the 2024 Korea Youth Risk Behavior Survey. Depressive symptoms were assessed using a single self-reported item. Weekday and weekend smartphone use (h/day) were modeled as continuous variables. Multivariable logistic regression analyses were conducted adjusting for sociodemographic factors, health behaviors, perceived stress, and anxiety symptoms. Nagelkerke pseudo-*R*^2^ was used to assess incremental explanatory value.

**Results:**

Weekday smartphone use showed a small positive association with depressive symptoms (OR = 1.02, 95% CI 1.01–1.03). In contrast, perceived loneliness demonstrated a strong graded association: moderate loneliness (OR = 2.54, 95% CI 2.40–2.68) and high loneliness (OR = 4.74, 95% CI 4.43–5.07) were associated with substantially higher odds of depressive symptoms. The inclusion of loneliness increased the model's explanatory power (ΔNagelkerke *R*^2^ = 0.163). Weekend smartphone use showed a marginal inverse association (OR = 0.99, 95% CI 0.98–1.00).

**Conclusions:**

Perceived loneliness showed a substantially stronger association with depressive symptoms than smartphone use duration. These findings highlight the importance of addressing social isolation in adolescent mental health interventions, alongside promoting balanced and context-sensitive smartphone use.

## 1 Introduction

Adolescent depression represents a significant global public health concern and is associated with increased suicide risk, impaired academic functioning, substance use, and adverse mental health outcomes in adulthood (1–3). A global systematic review and meta-analysis estimated that approximately 34% of adolescents experience elevated depressive symptoms, with prevalence increasing from 24% during 2001–2010 to 37% during 2011–2020 (4). These trends highlight the importance of identifying modifiable correlates associated with adolescent depressive symptoms.

In South Korea, nationally representative surveillance data indicate persistently high levels of depressive symptoms among adolescents. According to the 2024 Korea Youth Risk Behavior Survey, 27.7% of adolescents reported experiencing depressive symptoms in the past 12 months, with higher prevalence among girls (32.5%) than boys (23.1%) (5). In this highly digitalized social context, examining both digital behavior exposure and psychosocial vulnerabilities may provide important insights into adolescent mental health.

Evidence regarding the relationship between digital technology use and adolescent mental health remains mixed. Early studies suggested that increased screen time is associated with poorer psychological wellbeing (6). Meta-analytic findings indicate moderate associations between problematic smartphone use (PSU) and depression and anxiety (7). However, large-scale longitudinal and preregistered studies have reported generally small associations between daily screen time and mental health, often lacking clinical significance (8, 9). These findings suggest that duration of use alone may not fully explain variability in depressive symptoms.

Recent research has further emphasized that the impact of smartphone use on mental health may depend on contextual and qualitative factors rather than duration alone (10, 11). For example, distinctions between active (e.g., social interaction) and passive (e.g., content consumption) use, as well as differences in usage motivations, have been associated with divergent psychological outcomes (12). Moreover, emerging evidence suggests that excessive smartphone use may indirectly contribute to depressive symptoms through mechanisms such as sleep disruption, reduced face-to-face interaction, and attentional difficulties (13, 14). Taken together, these findings highlight the limitations of exposure-based models and underscore the importance of considering behavioral mechanisms underlying digital technology use.

In contrast, problematic smartphone use (PSU), characterized by behavioral dysregulation and functional impairment, has demonstrated stronger associations with depression in meta-analytic research, with pooled odds ratios exceeding 3.0 in some studies (15–17). This distinction underscores the methodological importance of differentiating between exposure duration and behavioral dysregulation.

Beyond digital behavior, loneliness has been consistently identified as a significant psychosocial correlate of depressive symptoms. Theoretical and empirical research suggests that loneliness influences emotional regulation and cognitive processing and may affect mental health through sleep disruption and stress-related pathways (18). Meta-analytic evidence indicates a moderate positive association between loneliness and depressive symptoms (19). A systematic review further reported that loneliness significantly increases the risk of depression among children and adolescents, with duration of loneliness exerting stronger effects than intensity (20).

In addition, recent studies suggest that loneliness and digital technology use may be dynamically interrelated (21). Adolescents experiencing loneliness may increase smartphone use as a compensatory strategy for social connection; however, such use may paradoxically reinforce perceived social isolation through processes such as social comparison and reduced offline interaction (22, 23). This bidirectional relationship indicates that loneliness may function not only as an independent risk factor but also as a potential mediator or moderator in the association between smartphone use and depressive symptoms (24).

Despite growing research interest in these interrelationships, evidence directly comparing the relative contributions of smartphone use duration and loneliness within a unified analytical framework remains limited, particularly in nationally representative samples. In the context of South Korea's highly digitalized society, further clarification of the relative roles of behavioral exposure (smartphone use duration) and psychosocial vulnerability (loneliness) may provide valuable evidence to inform more targeted and effective public health strategies for adolescent mental health.

Using nationally representative data from the 2024 Korea Youth Risk Behavior Survey, the present study aimed to: (1) examine the associations between weekday and weekend smartphone use duration and depressive symptoms; (2) assess the association between loneliness and depressive symptoms; and (3) compare the relative strength of these associations after adjusting for sociodemographic characteristics, health behaviors, and psychological covariates.

Based on the existing literature, we proposed the following hypotheses: (1) longer smartphone use duration will be associated with higher levels of depressive symptoms, although the magnitude of this association is expected to be modest; (2) higher levels of perceived loneliness will be strongly associated with increased odds of depressive symptoms; and (3) loneliness will demonstrate a stronger association with depressive symptoms than smartphone use duration after adjusting for relevant covariates.

## 2 Methods

### 2.1 Study design and data source

This study is a cross-sectional secondary analysis of data from the 20th Korea Youth Risk Behavior Survey (49). The KYRBS is an annual, nationally representative survey employing a stratified multistage cluster sampling design to monitor health behaviors among middle and high school students in South Korea. It is jointly conducted by the Korea Disease Control and Prevention Agency and the Ministry of Education (5).

The 2024 survey was conducted from June to July 2024 and covered all administrative regions of South Korea, including metropolitan cities and provinces, to ensure national representativeness. Schools were selected as primary sampling units, and classes were randomly selected within schools as secondary sampling units. Data were collected using an anonymous, self-administered, web-based questionnaire. Students completed the survey during regular school hours in computer classrooms using mobile devices (e.g., tablet PCs or smartphones) or computers under the supervision of trained teachers. Participation was voluntary, and responses were recorded electronically without personal identifiers to ensure confidentiality.

The survey protocol was approved by the Institutional Review Board of the Korea Disease Control and Prevention Agency (Approval No. 117058). Informed consent was obtained from all participants in accordance with national survey procedures.

In 2024, 54,653 students from 799 schools (400 middle schools and 399 high schools) participated, yielding a response rate of 94.9%. Participants with complete data on depressive symptoms were included in the present analysis (*N* = 54,653).

### 2.2 Measures

#### 2.2.1 Depressive symptoms

Depressive symptoms were assessed using a single item asking whether participants had experienced sadness or hopelessness lasting at least two consecutive weeks during the past 12 months that interfered with daily functioning (0 = no; 1 = yes) (5). This item has been widely used in national adolescent health surveillance.

#### 2.2.2 Smartphone use duration

Participants reported their average daily smartphone use (h/day) separately for weekdays and weekends during the past 7 days. Both variables were treated as continuous predictors in regression analyses. Students reporting no smartphone use were included in descriptive analyses but excluded from regression models to focus on dose–response associations among active users.

#### 2.2.3 Loneliness

Loneliness was assessed using a single item with five response categories (“never,” “rarely,” “sometimes,” “often,” “always”). Responses were recoded into three categories: low loneliness (“never/rarely,” reference group), moderate loneliness (“sometimes”), and high loneliness (“often/always”).

#### 2.2.4 Anxiety symptoms

Anxiety symptoms were measured using the Generalized Anxiety Disorder-7 (GAD-7) scale (25). Total scores range from 0 to 21, with higher scores indicating greater anxiety severity. Internal consistency in the present sample was excellent (Cronbach's α = 0.92). The total score was entered as a continuous variable.

#### 2.2.5 Perceived stress

Perceived stress was assessed using a single item with five response levels (“very high,” “high,” “moderate,” “low,” “none”) and recoded into three categories: low (reference), moderate, and high stress.

### 2.3 Covariate

Sociodemographic variables included sex (male/female), academic performance (high, middle, low), and subjective family economic status (high, middle, and low).

Health behavior variables included self-rated health (good, fair, poor), physical activity frequency (0 days/week, 1–3 days/week, ≥4 days/week), sleep recovery sufficiency (sufficient, moderate, insufficient), lifetime alcohol use (yes/no), and lifetime cigarette smoking (yes/no).

Categorical variables were entered into regression models using dummy coding, with the lowest-risk or theoretically normative category serving as the reference group.

### 2.4 Statistical analysis

All analyses were conducted using IBM SPSS Statistics for Windows, Version 26.0 (IBM Corp., Armonk, NY, USA). Analyses were conducted without applying sampling weights, as the primary aim was to examine associations between variables rather than to estimate population-level prevalence. This approach is consistent with prior epidemiological studies using KYRBS data. Two-tailed tests were applied, with statistical significance set at *p* < 0.05.

Descriptive statistics are presented as frequencies and percentages for categorical variables and mean (SD) for continuous variables. Group differences were examined using Pearson's chi-square tests for categorical variables and independent samples *t*-tests for continuous variables.

Five hierarchical binary logistic regression models were constructed, each building upon the previous one to assess incremental explanatory contributions:

Model 1: Weekday and weekend smartphone use duration;
Model 2: Model 1 + sociodemographic variables;
Model 3: Model 2 + health behavior variables;
Model 4: Model 3 + loneliness;
Model 5: Model 4 + perceived stress and GAD-7 total score.

Results are reported as odds ratios (ORs) with 95% confidence intervals (CIs).

Model explanatory power was evaluated using Nagelkerke's pseudo-*R*^2^. Changes in *R*^2^ (Δ*R*^2^) were calculated to assess incremental explanatory contributions across hierarchical models. Model fit was assessed using the Hosmer–Lemeshow goodness-of-fit test (*p* > 0.05 indicating acceptable fit). To address potential over-adjustment, a conceptual framework guided the hierarchical approach: sociodemographic and health behavior variables as distal determinants, loneliness as the proximal risk factor, and perceived stress and anxiety as shared pathway components. Multicollinearity was examined using variance inflation factors (VIF), with all values below 5 indicating no evidence of serious multicollinearity.

## 3 Results

### 3.1 Sample characteristics

A total of 54,653 adolescents were included in the analysis. Descriptive statistics for all study variables are presented in Table 1.

**Table 1:** Characteristics of the study sample (*N* = 54,653).

Variable	Category	*n*	%/Mean ±SD
Sociodemographic characteristics			
Sex	Male	28,090	51.4
	Female	26,563	48.6
School grade	Middle school	29,087	53.2
	High school	25,566	46.8
Academic performance	High	20,838	38.1
	Moderate	15,844	29.0
	Low	17,968	32.9
Subjective family economic status	Good	23,143	42.3
	Average	25,431	46.5
	Poor	6,074	11.1
Mental health characteristics			
Depressive symptoms	No	39,488	72.3
	Yes	15,165	27.7
GAD-7 total score	—	—	11.45 ± 4.74
Psychosocial factors			
Loneliness	Low	24,425	44.7
	Moderate	20,067	36.7
	High	10,161	18.6
Perceived stress	Low	31,611	57.8
	Moderate	16,902	30.9
	High	6,140	11.2
Health behaviors			
Self-rated health	Good	36,196	66.2
	Average	13,352	24.4
	Poor	5,105	9.3
Physical activity	0 days/week	16,367	29.9
	1–3 days/week	24,140	44.2
	≥4 days/week	14,146	25.9
Sleep recovery	Adequate	12,328	22.6
	Moderate	16,574	30.3
	Insufficient	25,751	47.1
Alcohol use	No	37,998	69.5
	Yes	16,655	30.5
Cigarette smoking	No	50,442	92.3
	Yes	4,211	7.7
Digital media use			
Smartphone use	Weekday	52,832	96.7
	Weekend	52,798	96.6
Smartphone use (h/day)	Weekday	—	4.33 ± 2.85
	Weekend	—	6.30 ± 3.85

SD, standard deviation; GAD-7, Generalized Anxiety Disorder-7.

Categorical variables are presented as frequencies and percentages (n, %), and continuous variables are presented as mean ± standard deviation (SD).

The sample was evenly distributed by sex (51.4% boys; 48.6% girls), with slightly more middle school than high school students (53.2 vs. 46.8%). Academic performance was reported as high (38.1%), moderate (29.0%), and low (32.9%). Perceived household economic status was categorized as high (42.3%), middle (46.5%), and low (11.1%).

Overall, 27.7% of participants reported depressive symptoms lasting at least two consecutive weeks during the past 12 months. Loneliness levels were distributed as low (44.7%), moderate (36.7%), and high (18.6%). Perceived stress was categorized as low (57.8%), moderate (30.9%), and high (11.2%). The mean GAD-7 score was 11.45 (SD = 4.74).

Regarding health behaviors, 66.2% reported good self-rated health, 24.4% fair, and 9.3% poor. Physical activity frequency was 0 days/week (29.9%), 1–3 days/week (44.2%), and ≥4 days/week (25.9%). Adequate sleep recovery was reported by 22.6%, while 47.1% reported insufficient recovery. Lifetime alcohol use and cigarette smoking were reported by 30.5 and 7.7%, respectively.

Smartphone use was reported by 96.7% of participants on weekdays and 96.6% on weekends. Mean daily use was 4.33 h (SD = 2.85) on weekdays and 6.30 h (SD = 3.85) on weekends.

### 3.2 Bivariate associations

Bivariate associations with depressive symptoms are presented in Table 2.

**Table 2:** Bivariate associations between study variables and depressive symptoms (*N* = 54,653).

Variable	Category/Measurement	No depressive symptoms	Depressive symptoms	*p*-value
Sex	Male	21,585 (76.8)	6,505 (23.2)	< 0.001
Female	17,903 (67.4)	8,660 (32.6)	
Academic performance	High	15,772 (75.7)	5,066 (24.3)	< 0.001
Moderate	11,657 (73.6)	4,187 (26.4)	
Low	12,057 (67.1)	5,911 (32.9)	
Subjective family economic status	Good	17,094 (73.9)	6,049 (26.1)	< 0.001
Average	18,644 (73.3)	6,787 (26.7)	
Poor	3,748 (61.7)	2,326 (38.3)	
Weekday smartphone use (h/day)	Mean ± SD	4.16 ± 2.71	4.77 ± 3.15	< 0.001
Weekend smartphone use (h/day)	Mean ± SD	6.08 ± 3.70	6.87 ± 4.15	< 0.001
Loneliness	Low	21,917 (89.7)	2,508 (10.3)	< 0.001
Moderate	13,814 (68.8)	6,253 (31.2)	
High	3,757 (37.0)	6,404 (63.0)	
Perceived stress	Low	26,869 (85.0)	4,742 (15.0)	< 0.001
Moderate	10,343 (61.2)	6,559 (38.8)	
High	2,276 (37.1)	3,864 (62.9)	
GAD-7 total score	Mean ± SD	10.14 ± 3.68	14.84 ± 5.47	< 0.001
Self-rated health	Good	28,000 (77.4)	8,196 (22.6)	< 0.001
Average	8,826 (66.1)	4,526 (33.9)	
Poor	2,662 (52.1)	2,443 (47.9)	
Physical activity	0 days/week	11,955 (73.0)	4,412 (27.0)	< 0.001
1–3 days/week	17,365 (71.9)	6,775 (28.1)	
≥4 days/week	10,168 (71.9)	3,978 (28.1)	
Sleep recovery	Adequate	10,151 (82.3)	2,177 (17.7)	< 0.001
Moderate	12,601 (76.0)	3,973 (24.0)	
Insufficient	16,736 (65.0)	9,015 (35.0)	
Alcohol use	No	28,730 (75.6)	9,268 (24.4)	< 0.001
Yes	10,758 (64.6)	5,897 (35.4)	
Cigarette smoking	No	37,157 (73.7)	13,285 (26.3)	< 0.001
Yes	2,331 (55.4)	1,880 (44.6)	

SD, standard deviation.

Categorical variables were compared using Pearson's chi-square tests, and continuous variables were compared using independent samples t-tests. Data are presented as n (%) or mean ± standard deviation (SD).

#### 3.2.1 Smartphone use

Adolescents with depressive symptoms reported significantly longer smartphone use on both weekdays (4.77 ± 3.15 vs. 4.16 ± 2.71 h, *p* < 0.001) and weekends (6.87 ± 4.15 vs. 6.08 ± 3.70 h, *p* < 0.001).

#### 3.2.2 Psychosocial factors

The prevalence of depressive symptoms increased across loneliness categories: 10.3% (low), 31.2% (moderate), and 63.0% (high; *p* < 0.001). A similar gradient was observed for perceived stress (15.0%, 38.8%, and 62.9%, respectively; *p* < 0.001). Adolescents with depressive symptoms had higher mean GAD-7 scores compared with those without symptoms (14.84 ± 5.47 vs. 10.14 ± 3.68, *p* < 0.001).

#### 3.2.3 Sociodemographic factors

Depressive symptoms were more prevalent among girls than boys (32.6 vs. 23.2%, *p* < 0.001). Lower academic performance and lower household economic status were associated with higher prevalence (both *p* < 0.001).

#### 3.2.4 Health behaviors

Poor self-rated health and insufficient sleep recovery were associated with higher prevalence of depressive symptoms (both *p* < 0.001). Physical activity frequency showed statistically significant differences across groups, although effect sizes were small. Lifetime alcohol use and cigarette smoking were also associated with higher prevalence (both *p* < 0.001).

Overall, adolescents with depressive symptoms exhibited higher levels of smartphone use, loneliness, perceived stress, and anxiety symptoms compared with those without depressive symptoms. In addition, depressive symptoms were more prevalent among females and among adolescents with lower academic performance, poorer perceived health, and unfavorable health behaviors. Collectively, these findings indicate that multiple psychosocial and behavioral factors are associated with depressive symptoms at the bivariate level.

### 3.3 Multivariable logistic regression analysis

Hierarchical logistic regression results are shown in Table 3.

**Table 3:** Hierarchical logistic regression of smartphone use and loneliness on depressive symptoms (*N* = 54,653).

Variable	Model 1 OR (95% CI)	Model 2 OR (95% CI)	Model 3 OR (95% CI)	Model 4 OR (95% CI)	Model 5 OR (95% CI)
Weekday smartphone use (per 1-h increase)	1.047 (1.037–1.058)	1.037 (1.027–1.048)	1.018 (1.008–1.029)	1.020 (1.008–1.031)	1.021 (1.009–1.033)
Weekend smartphone use (per 1-h increase)	1.025 (1.018–1.033)	1.015 (1.007–1.023)	1.009 (1.001–1.017)	0.995 (0.986–1.005)	0.990 (0.981–0.999)
Female vs. Male	—	1.557 (1.499–1.618)	1.412 (1.356–1.470)	1.234 (1.182–1.289)	1.210 (1.158–1.264)
Academic performance Moderate vs. High	—	1.087 (1.036–1.140)	1.046 (0.996–1.099)	1.018 (0.969–1.070)	1.004 (0.956–1.054)
Academic performance Low vs. High	—	1.331 (1.269–1.397)	1.242 (1.181–1.306)	1.158 (1.100–1.218)	1.105 (1.048–1.165)
Subjective family economic status Average vs. Good	—	1.084 (1.041–1.128)	1.053 (1.011–1.098)	1.019 (0.978–1.062)	1.007 (0.967–1.049)
Subjective family economic status Poor vs. Good	—	1.479 (1.389–1.574)	1.355 (1.271–1.444)	1.214 (1.138–1.295)	1.142 (1.069–1.221)
Self-rated health Average vs. Good	—	—	1.571 (1.500–1.645)	1.272 (1.213–1.336)	1.064 (1.012–1.118)
Self-rated health Poor vs. Good	—	—	2.550 (2.393–2.717)	1.712 (1.600–1.835)	1.138 (1.053–1.229)
Physical activity 1–3 days/week vs. 0	—	—	1.214 (1.158–1.273)	1.162 (1.109–1.218)	1.178 (1.122–1.237)
Physical activity ≥4 days/week vs. 0	—	—	1.474 (1.395–1.558)	1.383 (1.304–1.467)	1.418 (1.336–1.504)
Sleep recovery Moderate vs. Adequate	—	—	1.314 (1.231–1.402)	1.142 (1.068–1.221)	1.103 (1.033–1.178)
Sleep recovery Insufficient vs. Adequate	—	—	1.917 (1.813–2.026)	1.412 (1.330–1.500)	1.122 (1.052–1.196)
Alcohol use (yes vs. no)	—	—	1.415 (1.353–1.479)	1.279 (1.221–1.340)	1.292 (1.233–1.354)
Cigarette smoking (yes vs. no)	—	—	1.681 (1.565–1.805)	1.584 (1.466–1.712)	1.612 (1.492–1.742)
Loneliness Moderate vs. Low	—	—	—	3.498 (3.321–3.686)	2.535 (2.400–2.678)
Loneliness High vs. Low	—	—	—	11.454 (10.783–12.167)	4.737 (4.426–5.071)
Perceived stress Moderate vs. Low	—	—	—	—	1.630 (1.549–1.716)
Perceived stress High vs. Low	—	—	—	—	2.409 (2.234–2.599)
GAD-7 total score (per 1-point increase)	—	—	—	—	1.125 (1.119–1.132)
Nagelkerke pseudo-*R^2^*	0.014	0.038	0.110	0.273	0.345

OR, odds ratio; CI, confidence interval; GAD-7, Generalized Anxiety Disorder-7.

Results are presented as odds ratios (ORs) with 95% confidence intervals (CIs) from hierarchical binary logistic regression models. Continuous variables are expressed per one-unit increase.

Model 1: unadjusted model including weekday and weekend smartphone use.

Model 2: additionally adjusted for sociodemographic variables (sex, academic performance, and subjective family economic status).

Model 3: additionally adjusted for health behavior variables (self-rated health, physical activity, sleep recovery, alcohol use, and cigarette smoking).

Model 4: additionally adjusted for loneliness.

Model 5: additionally adjusted for perceived stress and GAD-7 total score.

Model 1 (crude model)

Each additional hour of weekday smartphone use was associated with higher odds of depressive symptoms (OR = 1.047, 95% CI: 1.037–1.058, *p* < 0.001). Weekend use was also positively associated (OR = 1.025, 95% CI: 1.018–1.033, *p* < 0.001). Nagelkerke *R*^2^ was 0.014.

Model 2 (adjusted for sociodemographic variables)

After adjustment, associations for weekday smartphone use (OR = 1.037, 95% CI: 1.027–1.048) and weekend smartphone use (OR = 1.015, 95% CI: 1.007–1.023) remained statistically significant. Female adolescents had higher odds of depressive symptoms compared with males (OR = 1.557, 95% CI: 1.499–1.618). Compared with high academic performance, moderate performance was associated with slightly higher odds (OR = 1.087, 95% CI: 1.036–1.140), and low performance showed a stronger association (OR = 1.331, 95% CI: 1.269–1.397). Similarly, compared with high economic status, average (OR = 1.084, 95% CI: 1.041–1.128) and low economic status (OR = 1.479, 95% CI: 1.389–1.574) were associated with higher odds of depressive symptoms. The Nagelkerke *R*^2^ increased to 0.038.

Model 3 (further adjusted for health behaviors)

Weekday smartphone use remained significantly associated (OR = 1.018, 95% CI: 1.008–1.029, *p* = 0.001), while the association for weekend use attenuated (OR = 1.009, 95% CI: 1.001–1.017, *p* = 0.024). Poor self-rated health was associated with higher odds of depressive symptoms (average: OR = 1.571, 95% CI: 1.500–1.645; poor: OR = 2.550, 95% CI: 2.393–2.717). Higher physical activity frequency was also associated with increased odds (1–3 days/week: OR = 1.214, 95% CI: 1.158–1.273; ≥4 days/week: OR = 1.474, 95% CI: 1.395–1.558). Insufficient sleep recovery (moderate: OR = 1.314, 95% CI: 1.231–1.402; insufficient: OR = 1.917, 95% CI: 1.813–2.026), alcohol use (OR = 1.415, 95% CI: 1.353–1.479), and cigarette smoking (OR = 1.681, 95% CI: 1.565–1.805) were independently associated with depressive symptoms. The Nagelkerke *R*^2^ increased to 0.110 (Δ*R*^2^ = 0.072).

Model 4 (addition of loneliness)

After including loneliness, the Nagelkerke *R*^2^ increased to 0.273 (Δ*R*^2^ = 0.163). Compared with low loneliness, moderate loneliness was associated with higher odds of depressive symptoms (OR = 3.498, 95% CI: 3.321–3.686), and high loneliness showed a substantially stronger association (OR = 11.454, 95% CI: 10.783–12.167; both *p* < 0.001). Weekday smartphone use remained statistically significant (OR = 1.020, 95% CI: 1.008–1.031), whereas weekend use was not significant (OR = 0.995, 95% CI: 0.986–1.005).

Model 5 (fully adjusted model)

In the fully adjusted model, weekday smartphone use remained independently associated with depressive symptoms (OR = 1.021, 95% CI: 1.009–1.033, *p* = 0.001). Weekend use showed a small inverse association (OR = 0.990, 95% CI: 0.981–0.999, *p* = 0.027).

Loneliness remained strongly associated with depressive symptoms. Compared with low loneliness, moderate loneliness was associated with higher odds (OR = 2.535, 95% CI: 2.400–2.678), and high loneliness with substantially higher odds (OR = 4.737, 95% CI: 4.426–5.071; both *p* < 0.001). Perceived stress also showed a graded association (moderate: OR = 1.630, 95% CI: 1.549–1.716; high: OR = 2.409, 95% CI: 2.234–2.599).

Each one-point increase in GAD-7 score was associated with higher odds of depressive symptoms (OR = 1.125, 95% CI: 1.119–1.132, *p* < 0.001). The final model showed acceptable fit (Nagelkerke *R*^2^ = 0.345). All variance inflation factors were below 5, indicating no evidence of problematic multicollinearity.

## 4 Discussion

### 4.1 Key findings

Using nationally representative data from 54,653 South Korean adolescents, this study examined the relative associations of smartphone use duration and perceived loneliness with depressive symptoms. Three primary findings were observed.

First, weekday smartphone use duration remained independently associated with depressive symptoms after full adjustment; however, the magnitude of association was small (OR = 1.02). This finding is consistent with longitudinal and meta-analytic evidence indicating that associations between screen time and depressive symptoms are generally modest in magnitude (8, 9). Evidence from large cohort studies further suggests that cross-sectional associations often attenuate after adjustment for psychosocial variables (26).

The observed effect size was considerably smaller than those reported for problematic smartphone use (PSU), for which pooled odds ratios exceeding 3.0 have been documented (15). This contrast underscores the importance of distinguishing between exposure duration and maladaptive or dysregulated patterns of use when interpreting digital behavior research.

Second, perceived loneliness demonstrated a markedly stronger association with depressive symptoms. Adolescents reporting high loneliness had 4.74-fold higher odds of depressive symptoms compared with those reporting low loneliness. Inclusion of loneliness substantially increased model explanatory power. These findings are consistent with theoretical frameworks suggesting that loneliness is associated with altered emotional regulation and threat-related cognitive processing (18). Longitudinal studies indicate that loneliness predicts subsequent depressive symptoms over time (20), and meta-analytic evidence demonstrates a moderate positive association in youth populations (19). Network analyses have further identified loneliness as closely connected to core affective symptoms of depression (25).

Third, a weekday–weekend dissociation pattern was observed. Weekday smartphone use showed a weak positive association with depressive symptoms, whereas weekend use demonstrated a small inverse association after full adjustment. This pattern may reflect contextual differences in digital engagement. The “Goldilocks hypothesis” suggests that moderate levels of digital engagement may not be uniformly detrimental and that associations with wellbeing may be non-linear (27). Weekday use may coincide with academic demands and sleep restriction, whereas weekend use may involve more discretionary or socially oriented activities. The inverse association for weekend smartphone use (OR = 0.99) was very close to the null and should be interpreted with caution. Given its trivial magnitude and marginal statistical significance, this finding may represent a chance association rather than a meaningful pattern. We therefore consider it exploratory and warranting replication in future studies.

### 4.2 Independent contribution of smartphone use duration

Overall, the findings align with growing evidence indicating that total screen time duration alone shows limited independent association with adolescent mental health outcomes. Longitudinal research generally reports small predictive effects for internalizing symptoms (8), whereas stronger associations are observed for problematic or compulsive use patterns (15, 16).

These findings suggest that quantitative exposure measures may not fully capture the complexity of digital behavior. Consideration of usage motivations, emotional regulation processes, and functional impairment may provide greater explanatory value than duration alone.

### 4.3 Loneliness as a key psychosocial correlate

Loneliness exhibited a robust and consistent association with depressive symptoms across models. Prior longitudinal research supports bidirectional associations between loneliness and depression (28, 29), suggesting dynamic interplay rather than unidirectional causality. Loneliness has also been proposed as a potential mediator in associations between problematic smartphone use and depressive symptoms (30).

In digitally mediated environments, online engagement may function both as a compensatory social resource and as an avoidance strategy that reinforces perceived isolation (31). These findings highlight the relevance of social connectedness in adolescent mental health research.

### 4.4 Sociodemographic and health behavior covariates

#### 4.4.1 Sex differences

Female adolescents had higher odds of depressive symptoms compared with males (OR = 1.19), consistent with epidemiological evidence (4, 32). Proposed mechanisms include differences in stress reactivity, neuroticism-related traits, and pubertal hormonal changes (33). Rumination has been identified as a potential psychological mechanism underlying sex disparities in depressive symptoms (34).

#### 4.4.2 Academic performance and socioeconomic status

Lower academic performance remained independently associated with depressive symptoms (OR = 1.34). Prior research suggests bidirectional associations between academic achievement and depressive symptoms (35, 36). Socioeconomic disadvantage has also been linked to adolescent depressive symptoms through pathways involving economic stress and family functioning (37–39).

#### 4.4.3 Self-rated health and sleep

Poor self-rated health and insufficient sleep restoration were independently associated with depressive symptoms. Sleep disturbance is a well-established correlate and potential antecedent of adolescent depression (40–42). Evidence also suggests reciprocal associations between depressive symptoms and subsequent physical health outcomes (43, 50).

#### 4.4.4 Physical activity

The positive association between physical activity frequency and depressive symptoms may reflect limitations of frequency-based measurement. Prior studies indicate that contextual characteristics—including activity type, intensity, and social setting—may be more relevant than frequency alone (44, 45). More granular assessment of activity characteristics may clarify this relationship.

#### 4.4.5 Substance use

Lifetime alcohol consumption (OR = 1.29) and cigarette smoking (OR = 1.61) were independently associated with depressive symptoms. Previous research suggests possible bidirectional associations between substance use and depression, although causal pathways remain to be established (46–48).

### 4.5 Strengths and limitations

This study has several strengths. It utilized a large nationally representative sample and incorporated comprehensive adjustment for sociodemographic and behavioral variables. The hierarchical modeling strategy allowed estimation of incremental explanatory contributions across conceptual domains.

Several limitations should be acknowledged. First, the cross-sectional design precludes causal inference and does not permit evaluation of temporal ordering. Accordingly, all interpretations are strictly confined to associations. Second, although the KYRBS employs a complex sampling design, sampling weights were not applied in regression analyses; therefore, caution is warranted when generalizing prevalence estimates. This limitation should be considered when interpreting the findings, although it is not expected to alter the primary conclusions regarding relative associations. Third, smartphone use was assessed solely by duration without capturing content, context, or purpose. Fourth, depressive symptoms were measured using a single self-report item, and all variables relied on self-report data, which may introduce reporting bias.

Taken together, these findings also have important implications for research, practice, and policy. The relatively small association between smartphone use duration and depressive symptoms suggests that interventions focusing exclusively on reducing screen time may have limited effectiveness. Instead, greater emphasis should be placed on promoting balanced and context-sensitive patterns of digital engagement. In educational and community settings, the strong association between loneliness and depressive symptoms highlights the importance of fostering social connectedness among adolescents. School-based programs that enhance peer relationships, social support, and emotional skills may be particularly beneficial, and routine screening for loneliness may help identify individuals at increased risk. In clinical contexts, loneliness may serve as a meaningful psychosocial indicator for early detection of depressive symptoms. Incorporating assessments of loneliness into routine mental health evaluations may improve identification and intervention strategies. Finally, the observed differences between weekday and weekend smartphone use underscore the importance of considering contextual and temporal patterns of digital behavior. A comprehensive approach that integrates behavioral, psychological, and social factors is likely to be more effective than interventions targeting single risk factors in isolation.

## 5 Conclusion

In this nationally representative sample of South Korean adolescents, perceived loneliness demonstrated a stronger association with depressive symptoms than smartphone use duration. Although weekday smartphone use was statistically associated with depressive symptoms, the magnitude of association was small.

These findings suggest that subjective social isolation may represent a more prominent psychosocial correlate of adolescent depressive symptoms than screen time duration alone. Future longitudinal research incorporating more detailed assessments of digital behavior, sleep, and social interaction is needed to clarify potential mechanisms and temporal dynamics.

## Data Availability

The original contributions presented in the study are included in the article/supplementary material, further inquiries can be directed to the corresponding author.

## References

[1] Thapar A Eyre O Patel V Brent D. Depression in young people. Lancet. (2022) 400:617–31. doi: 10.1016/S0140-6736(22)01012-1 35940184

[2] Erskine HE Patton G Degenhardt L Scott JG Moffitt TE Copeland WE . A heavy burden on young minds: the global burden of mental and substance use disorders in children and youth. Psychol Med. (2015) 45:1551–63. doi: 10.1017/S0033291714002888 25534496 PMC5922255

[3] Copeland WE Alaie I Jonsson U Shanahan L. Associations of childhood and adolescent depression with adult psychiatric and functional outcomes. J Am Acad Child Adolesc Psychiatry. (2021) 60:604–11. doi: 10.1016/j.jaac.2020.07.895 32758528 PMC8051642

[4] Shorey S Ng ED Wong CHJ. Global prevalence of depression and elevated depressive symptoms among adolescents: a systematic review and meta-analysis. Br J Clin Psychol. (2022) 61:287–305. doi: 10.1111/bjc.12333 34569066

[5] Korea Disease Control and Prevention Agency. Korea Youth Risk Behavior Survey (2024). Available online at: https://www.kdca.go.kr/yhs/ (Accessed October 15, 2025).

[6] Twenge JM Campbell WK. Associations between screen time and lower psychological well-being among children and adolescents: evidence from a population-based study. Prev Med Rep. (2018) 12:271–83. doi: 10.1016/j.pmedr.2018.10.003 30406005 PMC6214874

[7] Augner C Vlasak T Aichhorn W Barth A. The association between problematic smartphone use and symptoms of anxiety and depression: a meta-analysis. J Public Health. (2023) 45:193–201. doi: 10.1093/pubmed/fdab350 34585243

[8] Odgers CL Jensen MR. Annual research review: adolescent mental health in the digital age: facts, fears, and future directions. J Child Psychol Psychiatry. (2020) 61:336–48. doi: 10.1111/jcpp.13190 31951670 PMC8221420

[9] Tang S Werner-Seidler A Torok M Mackinnon A Christensen H. The relationship between screen time and mental health in young people: a systematic review of longitudinal studies. Clin Psychol Rev. (2021) 86:102021. doi: 10.1016/j.cpr.2021.102021 33798997

[10] Craggs A Ford TJ Orben A. Social media use and internalizing symptoms in clinical and community adolescent samples: a systematic review and meta-analysis. JAMA Pediatr. (2024) 178:814–22. doi: 10.1001/jamapediatrics.2024.2078 38913335 PMC11197453

[11] Winstone L Mars B Haworth CMA Heron J Kidger J. Adolescent social media user types and their mental health and well-being: results from a longitudinal survey of 13-14-year-olds in the United Kingdom. JCPP Adv. (2022) 2:e12071. doi: 10.1002/jcv2.12071 37431459 PMC10242896

[12] Jarman HK Marques MD McLean SA Slater A Paxton SJ. Motivations for social media use: associations with social media engagement and body satisfaction and well-being among adolescents. J Youth Adolesc. (2021) 50:2279–93. doi: 10.1007/s10964-020-01390-z 33475925

[13] Dibben GO Martin A Shore CB Johnstone A McMellon C Palmer V . Adolescents' interactive electronic device use, sleep and mental health: a systematic review of prospective studies. J Sleep Res. (2023) 32:e13899. doi: 10.1111/jsr.13899 37029099 PMC10909457

[14] Brosnan B Haszard JJ Meredith-Jones KA Wickham SR Galland BC Taylor RW. Screen use at bedtime and sleep duration and quality among youths. JAMA Pediatr. (2024) 178:1147–54. doi: 10.1001/jamapediatrics.2024.2914 39226046 PMC11372655

[15] Sohn SY Rees P Wildridge B Kalk NJ Carter B. Prevalence of problematic smartphone usage and associated mental health outcomes amongst children and young people: a systematic review, meta-analysis and GRADE of the evidence. BMC Psychiatry. (2019) 19:356. doi: 10.1186/s12888-019-2350-x 31779637 PMC6883663

[16] Elhai JD Dvorak RD Levine JC Hall BJ. Problematic smartphone use: a conceptual overview and systematic review of relations with anxiety and depression psychopathology. J Affect Disord. (2017) 207:251–9. doi: 10.1016/j.jad.2016.08.030 27736736

[17] Carter B Payne M Rees P Sohn SY Brown J Kalk NJ. A multi-school study in England to assess problematic smartphone usage and anxiety and depression. Acta Paediatr. (2024) 113:2240–8. doi: 10.1111/apa.17317 39084660

[18] Hawkley LC Cacioppo JT. Loneliness matters: a theoretical and empirical review of consequences and mechanisms. Ann Behav Med. (2010) 40:218–27. doi: 10.1007/s12160-010-9210-8 20652462 PMC3874845

[19] Dunn C Sicouri G. The relationship between loneliness and depressive symptoms in children and adolescents: a meta-analysis. Behav Change. (2022) 39:134–45. doi: 10.1017/bec.2022.13

[20] Loades ME Chatburn E Higson-Sweeney N Reynolds S Shafran R Brigden A . Rapid systematic review: the impact of social isolation and loneliness on the mental health of children and adolescents in the context of COVID-19. J Am Acad Child Adolesc Psychiatry. (2020) 59:1218–39.e3. doi: 10.1016/j.jaac.2020.05.009 32504808 PMC7267797

[21] MacDonald KB Schermer JA. Loneliness unlocked: associations with smartphone use and personality. Acta Psychol. (2021) 221:103454. doi: 10.1016/j.actpsy.2021.103454 34844066

[22] Xu XP Liu QQ Li ZH Yang WX. The mediating role of loneliness and the moderating role of gender between peer phubbing and adolescent mobile social media addiction. Int J Environ Res Public Health. (2022) 19:10176. doi: 10.3390/ijerph191610176 36011810 PMC9407745

[23] Kaya B Cenkseven Önder F. Ostracism and sense of coherence: the mediating role of social media addiction in adolescents. J Genet Psychol. (2025) 186:208–24. doi: 10.1080/00221325.2024.2413494 39387844

[24] Zhao C Ding H Du M Yu Y Chen JH Wu AM . The vicious cycle between loneliness and problematic smartphone use among adolescents: a random intercept cross-lagged panel model. J Youth Adolesc. (2024) 53:1428–40. doi: 10.1007/s10964-024-01974-z 38555341

[25] Grygiel P Dolata R Humenny G Muszyński M. Depressive symptoms and loneliness among early adolescents: a psychometric network analysis approach. J Child Psychol Psychiatry. (2024) 65:199–214. doi: 10.1111/jcpp.13876 37550521

[26] Nagata JM Lee CM Hur JO Baker FC. What we know about screen time and social media in early adolescence: a review of findings from the Adolescent Brain Cognitive Development Study. Curr Opin Pediatr. (2025) 37:357–64. doi: 10.1097/MOP.0000000000001462 40172268 PMC12259366

[27] Przybylski AK Weinstein N. A large-scale test of the Goldilocks hypothesis. Psychol Sci. (2017) 28:204–15. doi: 10.1177/0956797616678438 28085574

[28] Vanhalst J Klimstra TA Luyckx K Scholte RHJ Engels RCME Goossens L. The interplay of loneliness and depressive symptoms across adolescence. J Youth Adolesc. (2012) 41:776–87. doi: 10.1007/s10964-011-9726-7 22045508

[29] Sarman A Çiftci N. Relationship between smartphone addiction, loneliness, and depression in adolescents: a correlational structural equation modeling study. J Pediatr Nurs. (2024) 76:150–9. doi: 10.1016/j.pedn.2024.02.019 38402746

[30] Shi X Wang A Zhu Y. Longitudinal associations among smartphone addiction, loneliness, and depressive symptoms in college students. Addict Behav. (2023) 142:107676. doi: 10.1016/j.addbeh.2023.107676 36878182

[31] Nowland R Necka EA Cacioppo JT. Loneliness and social internet use: pathways to reconnection in a digital world? Perspect Psychol Sci. (2018) 13:70–87. doi: 10.1177/1745691617713052 28937910

[32] Liu C Pi X Liu B Zhang L Luo M Zhang Y . Network characteristics of emotional resilience, anxiety, and depression among Chinese adolescents. Front Psychiatry. (2025) 16:1651506. doi: 10.3389/fpsyt.2025.1651506 41035959 PMC12479411

[33] Parker G Brotchie H. Gender differences in depression. Int Rev Psychiatry. (2010) 22:429–36. doi: 10.3109/09540261.2010.492391 21047157

[34] Johnson DP Whisman MA. Gender differences in rumination. J Psychopathol Behav Assess. (2013) 35:275–84.

[35] Verboom CE Sijtsema JJ Verhulst FC Penninx BWJH Ormel J. Longitudinal associations between depressive problems and academic performance. Dev Psychol. (2014) 50:247–57. doi: 10.1037/a0032547 23566082

[36] Deng Y Cherian J Khan NUN Kumari K Sial MS Comite U . Family and academic stress and depression. Front Psychiatry. (2022) 13:869337. doi: 10.3389/fpsyt.2022.869337 35782431 PMC9243415

[37] Devenish B Hooley M Mellor D. The pathways between socioeconomic status and adolescent outcomes. Am J Community Psychol. (2017) 59:219–38. doi: 10.1002/ajcp.12115 28127777

[38] Goodman E Huang B Wade TJ Kahn RS. A multilevel analysis of the relation of socioeconomic status to adolescent depressive symptoms. J Pediatr. (2003) 143:451–6. doi: 10.1067/S0022-3476(03)00456-6 14571218

[39] Cao M Tian Y Lian S Yang X Zhou Z. Family socioeconomic status and adolescent depressive symptoms: a moderated mediation model. J Child Fam Stud. (2021) 30:2652–63. doi: 10.1007/s10826-021-02068-1

[40] Yang J Fu X Liao X Li Y. Association of problematic smartphone use with poor sleep quality, depression, and anxiety: a systematic review and meta-analysis. Psychiatry Res. (2020) 284:112686. doi: 10.1016/j.psychres.2019.112686 31757638

[41] Castiglione-Fontanellaz CEG Tarokh L. Sleep and adolescent depression. Clin Transl Neurosci. (2023) 8:3. doi: 10.3390/ctn8010003

[42] Lovato N Gradisar M. A meta-analysis and model of the relationship between sleep and depression in adolescents. Sleep Med Rev. (2014) 18:521–9. doi: 10.1016/j.smrv.2014.03.006 24857255

[43] Johnson D Dupuis G Piche J Clayborne Z Colman I. Adult mental health outcomes of adolescent depression. Depress Anxiety. (2018) 35:700–16. doi: 10.1002/da.22777 29878410

[44] Kandola A del Pozo Cruz B Hayes JF Stubbs B. Impact on adolescent mental health of replacing screen-use with exercise. J Affect Disord. (2022) 301:240–7. doi: 10.1016/j.jad.2021.12.064 34999126

[45] Zhang Y Wu X Tao S Li S Ma L Yu Y . Associations between screen time, physical activity, and depressive symptoms during COVID-19. Environ Health Prev Med. (2021) 26:107. doi: 10.1186/s12199-021-01025-0 34727892 PMC8562930

[46] Yue Y Hong L Guo L Gao X Deng J Huang J . Gender differences in the association between smoking, alcohol use and depressive symptoms. Sci Rep. (2015) 5:17959. doi: 10.1038/srep17959 26639938 PMC4671152

[47] Hammerton G Lewis G Heron J Fernandes G Hickman M Lewis G. Alcohol use and depression in young adulthood. Lancet Psychiatry. (2023) 10:490–8. doi: 10.1016/S2215-0366(23)00138-4 37271164 PMC10659986

[48] Farooqui M Shoaib S Afaq H Quadri S Zaina F Baig A . Bidirectionality of smoking and depression in adolescents: a systematic review. Trends Psychiatry Psychother. (2023) 45:e20210429. doi: 10.47626/2237-6089-2021-0429 35738567 PMC10416256

[49] Korea Disease Control and Prevention Agency Ministry Ministry of Education. The 20th Korea Youth Risk Behavior Survey (KYRBS), 2024. Cheongju: Korea Disease Control and Prevention Agency (2024). Available online at: https://www.kdca.go.kr/yhs/home.jsp (Accessed October 15, 2025).

[50] Keenan-Miller D Hammen CL Brennan PA. Health outcomes related to early adolescent depression. J Adolesc Health. (2007) 41:256–62. doi: 10.1016/j.jadohealth.2007.03.015 17707295 PMC2034364

